# How Emotion Strengthens the Recollective Experience: A Time-Dependent Hippocampal Process

**DOI:** 10.1371/journal.pone.0001068

**Published:** 2007-10-31

**Authors:** Tali Sharot, Mieke Verfaellie, Andrew P. Yonelinas

**Affiliations:** 1 Wellcome Trust Centre for Neuroimaging, Institute of Neurology, University College London, London, United Kingdom; 2 Memory Disorders Research Center, Boston Department of Veterans Affairs and Boston University School of Medicine, Boston, Massachusetts, United States of America; 3 Department of Psychology, University of California at Davis, Davis, California, United States of America; Harvard Medical School, United States of America

## Abstract

Emotion significantly strengthens the subjective recollective experience even when objective accuracy of the memory is not improved. Here, we examine if this modulation is related to the effect of emotion on hippocampal-dependent memory consolidation. Two critical predictions follow from this hypothesis. First, since consolidation is assumed to take time, the enhancement in the recollective experience for emotional compared to neutral memories should become more apparent following a delay. Second, if the emotion advantage is critically dependent on the hippocampus, then the effects should be reduced in amnesic patients with hippocampal damage. To test these predictions we examined the recollective experience for emotional and neutral photos at two retention intervals (Experiment 1), and in amnesics and controls (Experiment 2). Emotional memories were associated with an enhancement in the recollective experience that was greatest after a delay, whereas familiarity was not influenced by emotion. In amnesics with hippocampal damage the emotion effect on recollective experience was reduced. Surprisingly, however, these patients still showed a general memory advantage for emotional compared to neutral items, but this effect was manifest primarily as a facilitation of familiarity. The results support the consolidation hypothesis of recollective experience, but suggest that the effects of emotion on episodic memory are not exclusively hippocampally mediated. Rather, emotion may enhance recognition by facilitating familiarity when recollection is impaired due to hippocampal damage.

## Introduction

One of the primary ways by which emotion modulates memory is by intensifying the recollective experience associated with memory retrieval. Subjects report an enhancement in the vividness of emotional memories, as well as an increase in confidence, even when accuracy per se is not enhanced [1],[2]. For example, a study examining students' recollection of the events of September 11, 2001 found that the accuracy for these memories did not differ from memories for everyday events, in both cases declining over time. However, ratings of vividness, recollection, and belief in the accuracy of memory declined only for mundane memories [3]. Recently, brain-imaging studies have suggested a role for the amygdala in emotion's influence on the recollective experience [2],[4],[5].

Here, we examine if the subjective recollective advantage enjoyed by emotional memories is related to the effect of emotion on hippocampal dependent memory consolidation. A wealth of data [6]–[Bibr pone.0001068-McGaugh1] [8] indicates that neurohormonal changes in response to emotional events activate β-adrenergic receptors in the amygdala, which in turn enhances hippocampal-dependent memory consolidation [9]. The effect of emotion on memory consolidation raises the possibility that the subjective qualities of memory are enhanced by emotion via this mechanism. If this is the case, then since consolidation takes time, the enhancement in the recollective experience for emotional relative to neutral memories should increase following a delay. Second, if the emotion advantage is critically dependent on the hippocampus, then patients with hippocampal damage should not exhibit the intense recollective experience normally reported for emotional memories.

Most studies that have examined the recollective experience for emotional stimuli have tested memory at only one point in time [1],[2],[4],[5] leaving it unclear whether the recollective advantage for emotional materials is related to consolidation. We have recently reported findings suggesting that the recollective experience of emotional memories may benefit from a time-dependent process [10]. However, in that study the time-dependent enhancement of the recollective experience could not be dissociated from time-dependent improvements of overall memory accuracy. In Experiment 1 we examine emotion's modulation of the recollective experience immediately after encoding and 24hrs later.

In addition, because no previous studies have examined the recollective experience for emotional stimuli in amnesics, it is unclear whether the hippocampus is critical for producing the subjective recollection advantage of emotional stimuli. Past studies have shown that both the perception of emotional photos, as indicated by ratings of arousal and valence, and the enhancement in recognition accuracy with emotion, are intact in amnesic patients [11],[12]. These results suggest that the hippocampus is not necessary for enhanced memory accuracy of emotional events. It remains unknown, however, if the same is true for the emotional enhancement of the subjective experience of recollection, which has been shown to be somewhat independent of the enhancement in accuracy [1], [3]. In a study examining patients with left hippocampal and amygdala pathology, the severity of hippocampal pathology was found to predict subjective reports of recollection for neutral and emotional items alike, suggesting that the hippocampus is critical for effective encoding of emotional and neutral material [13]. However, since memory was only tested immediately after encoding, no effect of emotion on memory was detected in either controls or patients. In Experiment 2 we examine the recollective experience for emotional and neutral photos in amnesics and controls after a delay that is sufficient to produce an emotion advantage in memory.

## Results

Experiment 1. 35 subjects (17 males, 18 females) were presented with two different sets of 60 neutral photos and 60 emotional photos from the International Affective Photo Series (IAPS) on two consecutive days. Immediately after the encoding session on day two participants were given a surprise recognition test including all previously viewed photos and 120 new photos (60 emotional, 60 neutral). Subjective experience of recollection was measured both by measuring recognition confidence and by asking for remember/know judgments.

First, we examined the effects of emotion on overall recognition accuracy as measured using overall hits and false alarm rates (collapsing across R and K responses) to calculate d'. A 2 (stimuli: emotion, neutral) by 2 (time: immediate, 24 h) ANOVA revealed that the stimuli type did not affect overall accuracy (F(1,34) = 2.28, p>0.1). This is consistent with several previous studies that have reported no effect of emotion on overall memory accuracy [1]–[Bibr pone.0001068-Sharot1] [3],[13]. There was a main effect of time (F [1], [34] = 63.96, P<0.0001) characterized by greater overall recognition accuracy at immediate testing. Importantly, there was no interaction between stimuli type and time (F[1], [34] = 1.4, P>0.2).

To examine the effects of emotion on different types of recognition responses we calculated ‘emotional difference scores’ which were the proportion of emotional stimuli in a given condition eliciting a particular recognition response minus the proportion of neutral stimuli eliciting that response. Larger positive values of these difference scores indicate that emotion has a larger beneficial effect on responding. We first examined the effects of emotion on high and low confidence recognition responses to determine if the effects of emotion were observed for the most confidently recognized items as well as for the less confidently recognized items. Second, we examined the effects of emotion on remember and know responses to determine if emotion enhanced the subjective experience of remembering or if it influenced recognition in the absence of a feeling of recollection. Finally, we examined the effects of emotion on recollection and familiarity by conducting a model-based analysis of the confidence and remember/know responses. The raw scores for each condition and response type are included in **Table S1**.

**Figure 1:**
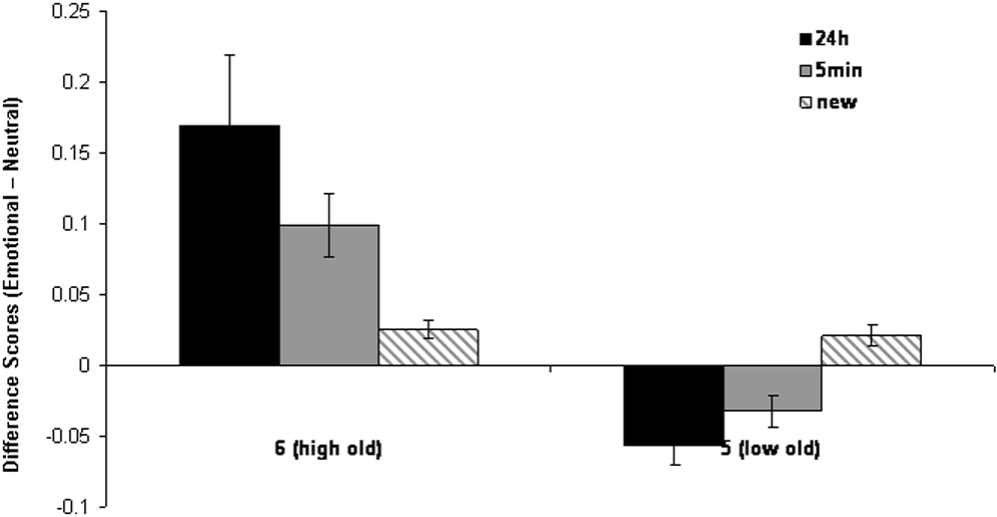
Differential effects of emotion on high and low confidence judgments over time. Confidence difference scores (Emotional-Neutral) for old stimuli encoded either 5 min or 24 h prior to recognition test, and for new stimuli, receiving either a 6 (high confidence that the stimuli is old), or 5 (low confidence that the stimuli is old) response. (error bars = sem).

**Figure 1** presents the effects of emotion on high and low confidence recognition responses. The figure indicates that the emotion effects were restricted to the high confidence recognition responses, and that they were larger for items studied 24 hours earlier than those studied 5 minutes earlier. To quantify these effects we conducted a 2 (time: immediate, 24 h) by 2 (confidence responses: 6, 5) ANOVA on the difference scores for old items. There was a significant interaction F [1], [34] = 9.5, P<0.005 that was characterized by a larger effect of emotion on high confidence old judgments after a 24h retention interval than immediately after encoding t [34] = 3.29, P<0.002, but no difference in emotion's effect on low confidence judgments over time. There were no significant effects on responses to new items.

**Figure 2:**
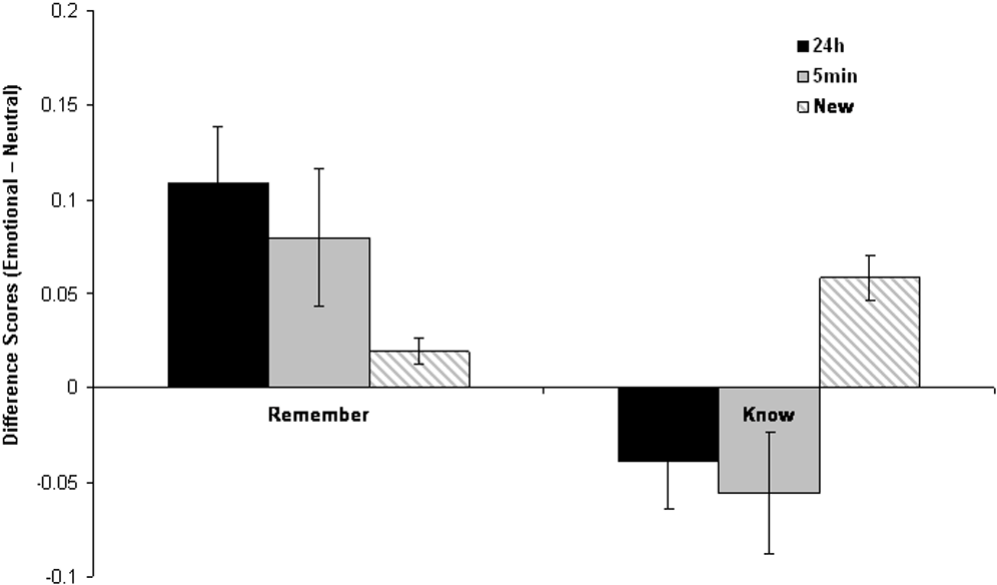
Differential effects of emotion on “remember' and “know” judgments over time. Difference scores (Emotional-Neutral) for old stimuli encoded either 5 min or 24 h prior to recognition test, and for new stimuli, receiving either a “remember” or “know” judgment. (error bars = sem).

**Figure 2** presents the effects of emotion on remember and know responses, and indicates that the emotion effects were observed for remember but not know responses, and that the effect of emotion was larger after a delay. To quantify these effects we conducted a 2 (time: immediate, 24 h) by 2 (response: remember, know) ANOVA on the difference scores for old items. There was a main effect of response F [34] = 6.08 P<0.05 which was characterized by a greater effect of emotion on “remember” responses than “know” responses. There was also a main effect of time which was characterized by a greater effect of emotion on old items tested after a 24 hour delay compared to those tested after a 5 minutes delay F [34] = 8.34 P<0.01. There was no interaction. For new items, there was a larger emotion effect for know than for remember responses, t [34] = 2.51 P<0.02, which reflected a general response bias to respond ‘know’ to new emotional items than new neutral items.

To further quantify the effects of emotion on recognition performance, we separated the effects of emotion on recollection and familiarity-based responses. Recollection and familiarity were estimated separately using the “remember/know” judgments and the recognition confidence judgments. First, recollection was measured as the proportion of old items receiving a remember response minus the proportion of new items receiving this response. Familiarity was measured as the probability of receiving a Know response given that a stimulus did not receive a Remember response, corrected for false alarms: K = (Khit/(1-Rhit))-(Kfa/(1-Rfa) [14]. Second, confidence-based receiver operating characteristics (ROCs) were plotted as a function of response confidence [14], and estimates of recollection and familiarity were derived using a least-squares method [15]. The model equation, P(‘old’|old) = P(‘old’|new)+R+(1-R) Φ (d'/2-ci)-Φ (-d'/2-ci), assumes that recognition reflects the contribution of recollection (R) and a signal detection based familiarity process. The variable d' reflects the distance between two equal-variance Gaussian strength distributions, ci reflects the response criterion at point i, and Φ is the cumulative normal response function. To facilitate the comparison to recollection, which was measured as a probability, each d' value was converted to the probability of a hit given a false alarm rate of 0.10.

**Figure 3:**
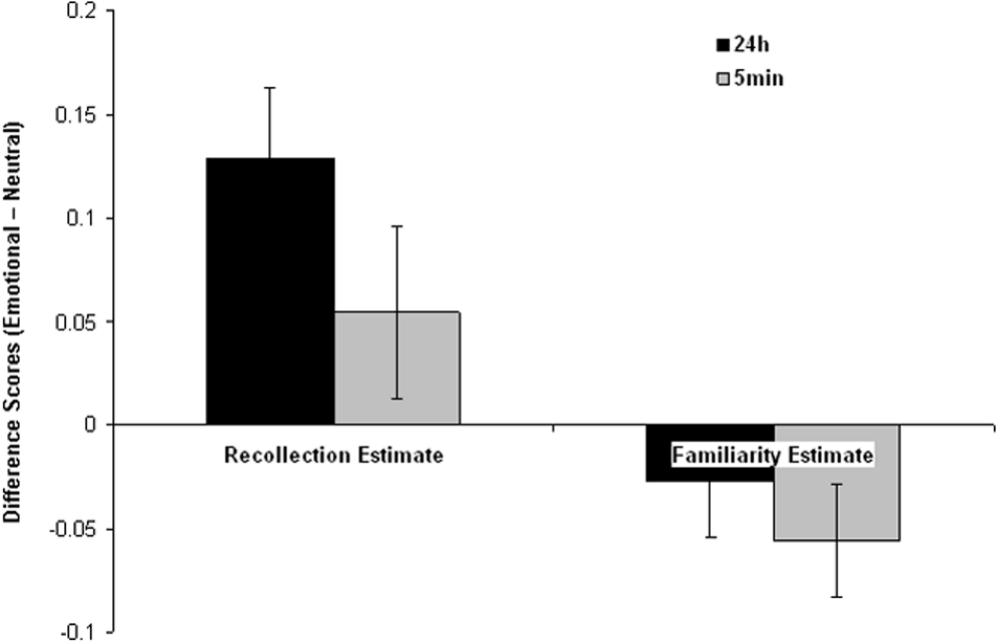
Differential effects of emotion on recollection and familiarity estimates over time. Difference scores (Emotional-Neutral) for estimates of recollection and familiarity (see method for details) for old stimuli encoded either 5 min or 24 h prior to recognition test, and for new stimuli. (error bars = sem).

To examine the effect of emotion on recollection and familiarity-based responses we calculated the difference scores by subtracting the recollection and familiarity estimates for neutral stimuli from that of emotional stimuli for each participant and condition. Scores were subjected to a 2 (response: recollection, familiarity) by 2 (time: immediate, 24 h) by 2 (procedure: R/K, ROC) ANOVA. There was a main effect of response (F [1], [34] = 8.45, P<0.01) that was characterized by a larger effect of emotion on recollection estimates than familiarity estimates. There was also a main effect of time (F [1], [34] = 14.46, P<0.001), which was characterized by a larger effect of emotion on memory after 24 hours than immediately after encoding (**Fig. 3**). These results are consistent with the analysis of the confidence and remember/know responses and indicate that emotion selectively enhanced recollection-based judgments in this task, and affects memory to a greater degree after a time delay.

**Figure 4:**
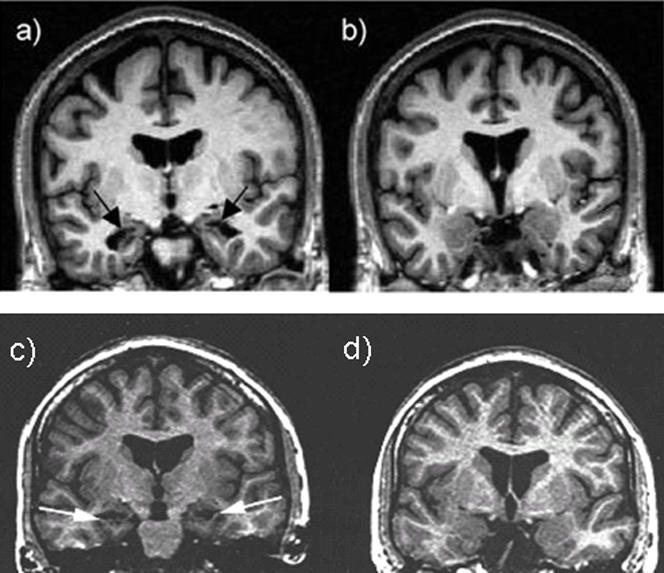
Anatomical images of amnesic patients. -T1 weighted coronal MRI sections of two of the patients35,36. Images reveal selective bilateral atrophy of hippocampus (a & d), and intact amygdala (b & c).

Experiment II . Nineteen healthy controls (nine males, ten females) and five amnesic patients (three males, two females) with bilateral hippocampal damage (either confirmed by volumetric analysis of structural magnetic resonance imaging (MRI) (Fig. 4) or suspected from etiological and behavioral profiles) were presented with 60 neutral photos and 60 emotional photos from the International Affective Photo Series (IAPS). Two hours later they were given a surprise recognition test including all previously viewed photos and 120 new photos (60 emotional, 60 neutral). Subjective experience of recollection was measured as in Experiment 1.

The results were analyzed in the same manner as in Experiment 1. The raw scores for each condition and response type are included in **Table S2**. We first examined the effects of emotion on overall recognition accuracy as measured using overall hits and false alarm rates (collapsing across R and K responses) for emotional and neutral stimuli. A 2 (stimuli: emotional, neutral) by 2 (group: patient, controls) ANOVA did not reveal a group by stimuli type interaction (F[1], [22] = 1.4, P>0.7), nor significant main effects of stimuli type (F[1], [22] = 2.58, P>0.1) or group (F[1], [22] = 2.74, P>.01). This replicates previous studies showing that amnesics do not differ from controls with regards to the effect of emotion on overall recognition accuracy [11],[12], and indicates that any differences in the effects of emotion on remember/know and confidence recognition responses will not be due to differences in overall sensitivity to the emotion manipulation.

**Figure 5:**
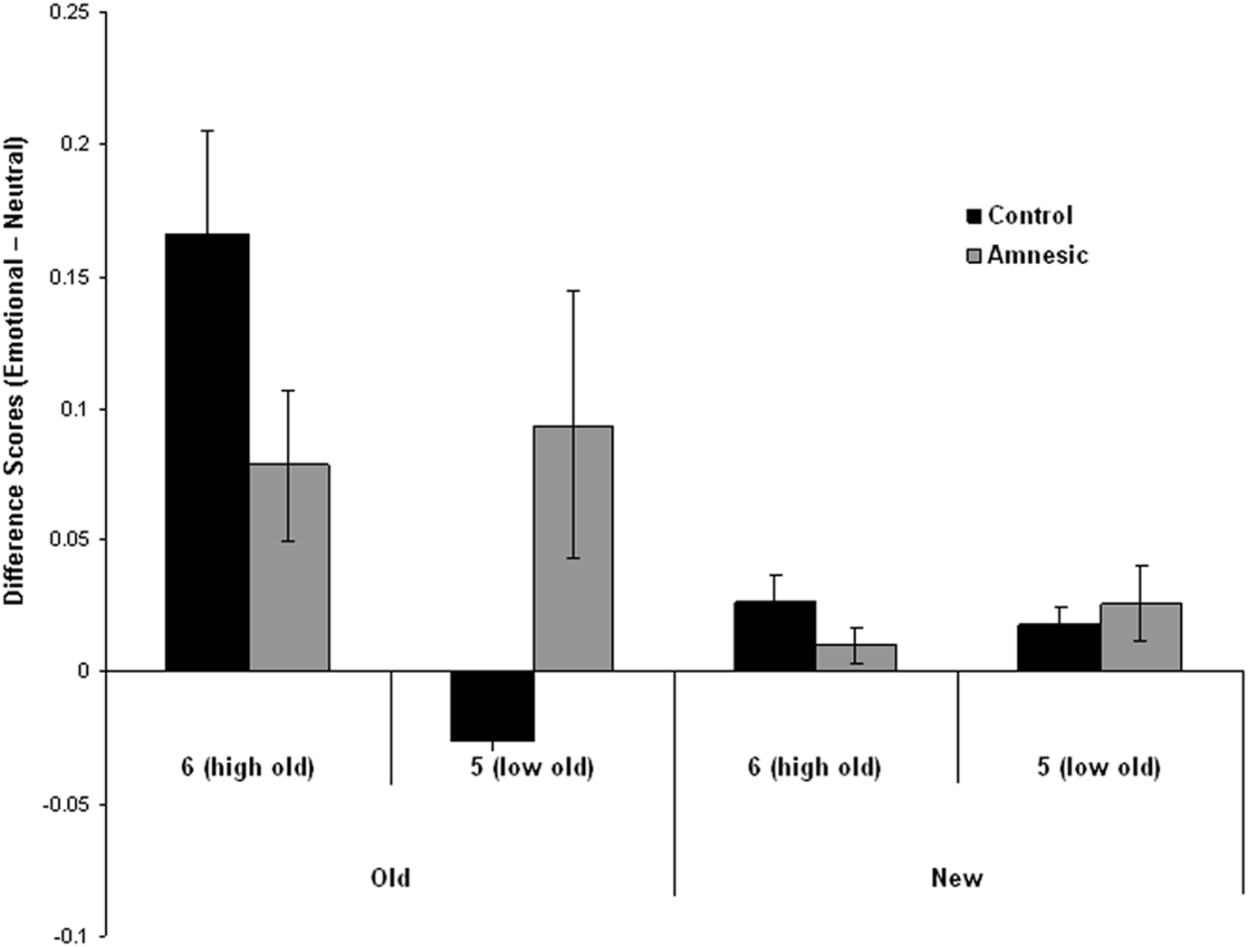
Differential effects of emotion on high and low confidence judgments in amnesics and controls. Confidence difference scores (Emotional-Neutral) for the amnesic and control groups, for old and new stimuli receiving either a 6 (high confidence that the stimuli is old), or 5 (low confidence that the stimuli is old) response. (error bars = sem).

**Figure 5** presents the effects of emotion on high and low confidence recognition responses. The figure indicates that the control subjects exhibited an emotion effect only for high confidence responses. In contrast, the amnesics exhibited a reduced emotion effect on high confidence responses, and they showed an emotion effect on low confidence recognition responses. To quantify these effects we conducted a 2 (confidence responses: 6, 5) by 2 (group: amnesic, control) ANOVA on the difference scores of old photos. There was a confidence by group interaction F [1], [22] = 4.41, P<0.05 that arose because in the control subjects the emotion advantage was significantly greater for high confidence responses than low confidence responses t [18] = 4.09, P<0.001, whereas for the patients, emotion had comparable effects on high and low confidence responses t [4] = .22, P>0.8. Furthermore, the control subjects tended to have a greater effect of emotion on high confidence judgments than the patients t [22] = 1.78, P = 0.09, while the patients tended to have a greater effect of emotion on low confidence judgments than the controls t [22] = 2.27, P = 0.07. There were no significant effects on the responses to new items.

**Figure 6:**
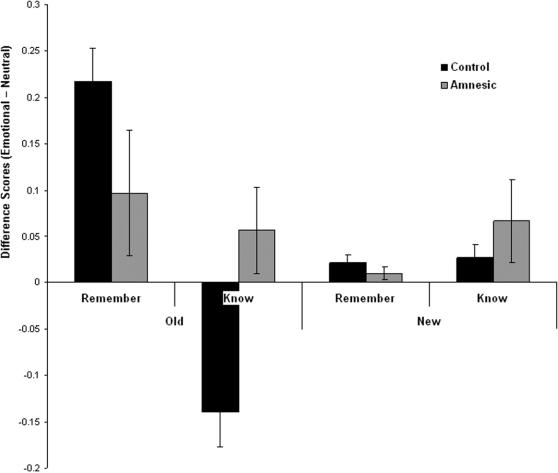
Differential effects of emotion on “remember' and “know” judgments in amnesics and controls. Difference scores (Emotional-Neutral) for the amnesic and control groups, for old and new stimuli receiving either a “remember” or “know” judgment. (error bars = sem).”

**Figure 6** presents the emotion effects for remember and know responses. The figure indicates that in control subjects only the remember responses exhibited an emotion advantage, whereas for the amnesics both remember and know responses exhibited an emotion advantage. A 2 (response: remember, know) by 2 (group: control, amnesic) ANOVA on the difference scores for old items revealed an interaction F [1], [22] = 5.17, P<0.05 that arose because in the control subjects the emotion advantage was significantly greater for remember responses than know responses t [18] = 5.32, P<0.0001, whereas for the patients emotion had comparable effects on remember and know responses t [4] = 0.45, P>0.6. Furthermore, the patients showed a greater emotional effect on know responses than the controls t [22] = 2.5, P<0.02. There were no significant effects on responses to new items.

**Figure 7:**
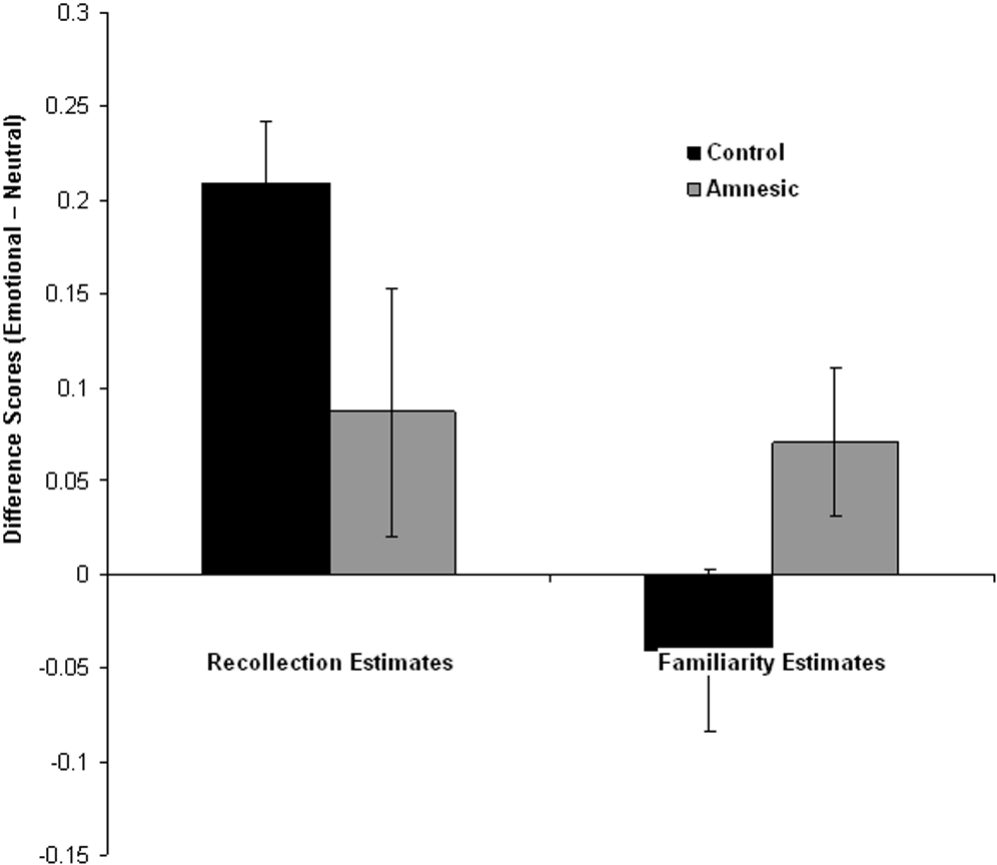
Differential effects of emotion on recollection and familiarity estimates in amnesics and controls. Difference scores (Emotional-Neutral) for the amnesic and control groups, for estimates of recollection and familiarity (see method for details). (error bars = sem).

As in Exp 1 estimates of recollection and familiarity were derived based on remember/know judgments. ROC estimates were not included because two patients' ROCs did not span a sufficient range to support parameter estimation. A 2 (response: recollection, familiarity) by 2 (group: control, amnesic) ANOVA revealed a significant interaction F [1], [22] = 4.46, P<0.05 that arose because in the control subjects the emotion advantage was significantly greater for recollection estimates than familiarity estimates t [18] = 6.35, P<0.0001, whereas for the patients emotion had comparable effects on recollection and familiarity estimates t [4] = 1.29, P>0.2 (**Fig. 7**). These results are consistent with the analysis of the confidence and remember/know responses and indicate that in controls emotion selectively enhanced recollection-based judgments, whereas in the amnesics a comparable emotion advantage was observed in both recollection and familiarity-based responses.

## Discussion

The current experiments reveal several findings that are crucial for understanding how emotion shapes memory for prior events. First, we show that the relative enhancement in the recollective experience associated with emotion benefits from the operation of a time-dependent process, consistent with consolidation. Moreover, the advantage in recognition memory related to emotion is experienced as recollection in healthy subjects. However, in amnesics the beneficial effects of emotion on recollection was reduced, and emotion began to enhance familiarity based recognition responses.

In both experiments we measured the subjective recollective experience by measuring recognition confidence and by asking for remember/know judgments. Aside from providing indexes of two critical aspects of the recollective experience the methods were also used to derive model-based estimates of recollection and familiarity-two memory process thought to underlie recognition performance [16]–[Bibr pone.0001068-Mandler1] [18]. “Remember” and high confidence judgments are thought to reflect recollection-based judgments, while “know” and low-confidence judgments are thought to be related to familiarity-based recognition judgments [18],[19]. Importantly, regardless of whether a model-based analysis (i.e. estimates of recollection and familiarity) or a model-free analysis (i.e. proportion of specific responses) was used, and whether confidence judgments or remember/know judgments were examined, the results converged on the same conclusions.

In Experiment I recognition confidence judgments and remember/know reports were collected at two retention intervals. The findings showed that the relative enhancement in the recollective experience of emotional memories compared with neutral memories was larger after a delay. Although emotion's effects on attention and perception during encoding [20],[21] may boost the recollective experience immediately [22],[23], the present findings indicate that the relative emotional advantage increases over time. The results of Experiment I are consistent with previous results from our lab [10] and extend them in several important ways. First, we show that the time-depended effects of emotion on the recollective experience are not contingent on time-dependent improvements on overall recognition accuracy. Second, we show these effects using two different measures of the recollective experience, demonstrating that the results are not a by-product of one specific paradigm. The result suggests that emotion elicits a mechanism that modulates memory retention, resulting in greater vividness and confidence of memories for emotional events relative to memories of neutral events after a delay. This is consistent with the notion that the emotional advantage in the recollective experience is due in part to slower forgetting, and with the suggestion that the sluggish consolidation of memories serves an adaptive function by enabling neurohormonal processes trigged by an arousing stimulus to modulate memory strength over time [9].

Experiment 2 verified that in healthy individuals recollection-based judgments, but not familiarity-based judgments, were heightened with emotion [also see 1], [2], [4]. However, in amnesia the emotional enhancement of recollection was reduced, and the effect of emotion on recognition judgments began to manifest itself as a facilitation of familiarity. As a result the patients exhibited a larger emotional advantage in familiarity-based recognition judgments than the controls. Past studies of amnesics with hippocampal damage have found a normal effect of emotion on recognition accuracy [11],[12]. The current findings are consistent with these results, but they reveal that emotion does not influence recognition in the same way as in healthy subjects. Whereas in healthy subjects the effects of emotion extend mostly to recollection, impaired function of the hippocampus proper still allows preferential recognition of emotional material by way of familiarity in the absence of recollection. Thus, an intact hippocampus may not be necessary in order to lead to a normal overall memory advantage for emotional compared to neutral items, because when the hippocampus is damaged the emotional advantage that usually affects recollection now benefits familiarity-based recognition.

The effects of emotion on recognition observed here can be interpreted within dual process and single process theories of recognition memory. Although the study was not designed to differentiate amongst those classes of models it is useful to consider the results in light of those theoretical approaches. From the perspective of dual process models the current results are consistent with previous work indicating that it is the process of recollection that is particularly sensitive to the effects of emotion [1],[2],[4]. The current results further indicate that these beneficial effects of emotion are time dependent, as expected if this process is involved in consolidation. In addition, the results indicate that the effects of emotion are not limited to recollection in the sense that when recollection was disrupted by amnesia emotion began to affect familiarity. We speculate that emotion will also enhance familiarity-based recognition in healthy individuals whenever recollection is unavailable, for example when attention is limited.

In contrast, by single process models in which recognition is assumed to be based on a global measure of memory strength, the results suggest that the effects of emotion on recognition memory can be observed even when emotion does not have a pronounced effect on overall memory strength. That is, the d' analysis of recognition accuracy indicated that emotion did not have large effects on overall recognition sensitivity. Thus, the observation that emotional items began to attract more remember and high confidence responses than nonemotional items, and that the effect of emotion on remembering was altered in amnesia can't be explained as reflecting differences in overall memory strength. Rather these differences must have occurred for different reasons such as shifts in response criterion for emotional compared to neutral items, or increases in the relative variance of the emotional items compared to neutral items. One possibility is that consolidation may preserve the memory strength of some emotional items more so than others, thus giving rise to a relative increased variance for the studied emotional compared to neutral items. In any case, consistent with the dual process account, it is clear that the effects of emotion on recollective experience, as measured by reports of remembering and high confidence responses, are significantly modulated by delay and by medial temporal lobe damage. Future studies examining autobiographical memories are needed to verify that these effects generalize to “real-life” situations.

On the basis of the current results, we speculate that the amygdala supports a time-dependent consolidation of emotional memories, but that the manner in which it contributes to recognition depends on the availability of recollection and familiarity signals from other medial temporal lobe regions. Namely, when recollective information from the hippocampus is available, memory associated with emotional aspects of the event is experienced as recollection, whereas under conditions in which recollection is compromised, performance relies more on familiarity information originating from other regions such as the rhinal cortex [24]. Although future studies are required to determine the precise neural mechanism by which emotion facilitates familiarity when recollection is impaired, studies in non-human animals have identified a neural pathway that may underlie such facilitation. Specifically, strong bi-directional projections between the amygdala and the rhinal cortices (i.e perirhinal and entorhinal cortices) have been identified in non-human primates [25]. Furthermore, studies in cats suggest that strength of the interaction between the perirhinal and entorhinal cortices may be increased by amygdala activity [26]. The rhinal cortices make up the chief path for impulse traffic to and from the hippocampus. It is possible that in the absence of incoming recollective signals from the hippocampus to the rhinal cortices and amygdala, the connectivity between the amygdala and rhinal cortices is strengthened, possibly facilitating familiarity. This is also consistent with a previous brain imaging study suggesting that amygdala activity mediates familiarity of stimuli previously experienced in an emotional context [27].

One should note that in three of our amnesic patients, bilateral ischemic damage to the hippocampus with sparing of adjacent areas could not be confirmed by MRI. These were cardiac arrest patients that suffered a brief period of hypoxia and had defibrillators that prevent the use of high resolution brain scans to quantify the cortical atrophy. The hippocampus is one of the brain regions that is particularly vulnerable to hypoxic-ischamic damage [28],[29]. However, other brain regions can also be affected, including the thalamus and the watershed regions in the cerebral cortex and cerebellum [30]. It is therefore impossible to determine with complete certainty which brain regions are affected in these patients. However, in cases in which the cognitive impairments are limited mainly to memory, as in the current patients, postmortem neuropathological analysis [28].[31] and volumetric neuroimaging [32] show that the hippocampus is the primary structure influenced by hypoxia and is the most probable source of the memory deficits. Furthermore, the behavioral profiles of the hypoxic patients for whom MRI scans were not available were similar to those of the hypoxic patients for whom bi-lateral damage confined to the hippocampus was confirmed by MRI, suggesting similar damage in both cases. Thus, the data suggests that the hippocampus is involved in the enhancement in the feeling of recollection, but not familiarity, of emotional stimuli.

In sum, our results suggest that the advantage in the recollective experience related to emotion is supported by a time-dependent mechanism, consistent with consolidation. However, although the hippocampus plays a role in strengthening the recollective experience for emotional events, the effects of emotion on recognition are not exclusively hippocampally mediated. Rather, emotion may enhance recognition by facilitating nonrecollective recognition when recollection is disrupted due to hippocampal damage.

## Materials and Methods

Experiment I: 35 undergraduate students at the University of California Davis participated in the study (age range 18 to 22; 18 females). All participants gave informed consent and received course credit for their participation. The study was approved by the committee on human research at the University of California.

An ANOVA (time×difference scores×gender) conducted to examine the influence of gender on the results did not reveal a significant interaction on confidence scores [F [2], [33] = 0.27, P = 0.77], R/K judgments [F [2], [33] = 0.04, P = 0.96], or ROC scores [F [2], [33] = 0.74, P = 0.49].

Stimuli consisted of 180 negatively arousing photos, and 180 neutral photos, selected from the International Affective Photo Series (IAPS), based on their standard scores for emotional arousal and emotional valence [33], and from our own set of neutral pictures to equate the two sets for the presence of humans and visual complexity [2]. Photos were rated in a previous study for valence and arousal [2]. Valence was rated on a scale from 1 (positive) to 9 (negative). Neutral photos were rated as neutral (mean = 3.75, SD = 1.07) and emotional photos as negative (mean = 7.69, SD = 0.52); t [10] = 14.23, P<0.0001. Arousal was rated on a scale from 1(not at all arousing) to 9 (very much arousing). Neutral photos had lower arousal ratings (mean = 3.03, SD = 0.83), than emotional photos (mean = 6.79, SD = 1.15); t [11]) = 10.67, P<0.0001.

The arousing negative photos had more categorical overlap than the neutral photos (such as mutated bodies). However, this difference can not explain our findings because the current paradigm was designed to look at changes in the effect of emotion on memory in different conditions (retention time and group). The categorical overlap exists in all these conditions (immediate and delay testing, amnesics and controls) and thus will be subtracted out in the analysis.

Participants viewed stimuli in two sessions separated approximately by 24 hs. In each session participants were presented with 60 neutral photos and 60 emotional photos. Different sets of emotional and neutral photos were presented on each day. Sets were counterbalanced across participants. On each trial a photo was presented for 1 s, after which the participant had 2 s to rate the photo for visual complexity, then a fixation cross appeared for 6 s. The trials were separated into four blocks of 30 trials each.

The subjective experience of recollection was measured both by measuring recognition confidence and by asking for remember/know judgments [19]. Immediately after the encoding session on day two, participants were trained to make remember/know judgments [34]; after reading the detailed instructions they were asked to explain the instructions in their own words, and were then given practice trails to verify that they fully understood the difference between a “remember” and “know” judgment. During the practice trials participants were asked to verbally justify their “remember” responses. Once the experimenter was assured that the participants understood and were following instructions (i.e. “remembered” stimuli were ones that evoked a specific memory for the episodic context in which the stimuli was experienced, such as a thought, feeling, or sensory detail) they were given the recognition test. The recognition test included the presentation of 360 photos: 60 old negatively arousing photos presented the previous day (day 1), 60 old negatively arousing photos presented that day (day 2), 60 old neutral photos presented on day 1, 60 old neutral photos presented on day 2, 60 new negatively arousing photos, and 60 new neutral photos. Thus, memory was tested both for photos presented a few minutes prior to the recognition test, and those presented 24 hrs earlier. Stimuli were presented in a random order on a computer screen. Each trial consisted of the presentation of a photo for 2 s, followed by 3 s to indicate whether the photo was new, “remembered”, or “known”, by pressing the appropriate key. Then the participants had 3 s to rate the confidence of their recognition response on a scale from 1 to 6. A ‘6’ response indicated that they were sure it was studied, a ‘5’ indicated they were unsure it was studied, and a ‘4’ indicated that they were guessing that it was studied. A ‘1’ response indicated that they were sure it was not studied, a ‘2’ indicated they were unsure it was not studied, and ‘3’ indicated that they were guessing that it was not studied.

Experiment 2: Nineteen healthy controls (nine males) and five patients (three males) participated in the study. The controls were selected to be matched in age (controls = 52.39, patients = 53.6, P>.05), and education (controls = 15.29, patients = 15, P>.05) to the patient group. Bilateral ischemic damage to the hippocampus was confirmed by structural magnetic resonance imaging (MRI), with sparing of adjacent medial temporal lobe structures, including the amygdala, in two patients (Fig. 4). Patient AM1 suffered an ischemic episode several years prior to testing with no other significant neurological history [35]. Patient AM2 had an asthma attack fifteen years prior to testing, followed by grand mal seizure. Together, these led to an anoxic encephalopathy that led to a dense anterograde amnesia [36]. Both their intelligence quotients (109, 100), as well as their performance on the Wisconsin Card Sorting Test (six categories each) were normal, but they were severely impaired on tests of long-term memory (both had WMS-R delay scores<50). The rest of the patients were cardiac arrest patients who suffered a brief period of hypoxia associated with coma with no prior history of brain pathology. The patients had defibrillators, which prevented the use of high-resolution brain scans to quantify the cortical atrophy. Bilateral hippocampus damage was suspected from etiological and behavioral profiles [37]. With the exception of memory impairments, the patients were cognitively intact. Their intelligence quotients (100, 110 and 119) were normal. Moreover, on the WMS-R they were normal on the attentional subscale (97, 100 and 125), but impaired on the delayed memory subscale (87, 77 and 77). All participants gave informed consent and were paid for their participation. The study was approved by the committee on human research at the University of California.

Estimates of medial temporal lobe damage for patients AM1 and AM2 were based on quantitative analysis of magnetic resonance images, as reported previously [38], in which volumes for each patient were compared to those obtained of 4 age- and gender-matched control subjects. The medial temporal structures were segmented individually as described previously [39]. The parahippocampal gyrus was defined anteriorly by the isthmus of the temporal and frontal lobes, medially by the collateral fissure, laterally by the hippocampal fissure, and posteriorly by the anterior limit of the calcarine fissure. A computer program, XVOL, was used to determine the volumes of the units of interest. The volumes obtained for each unit were summated for all slices in which each unit appeared. Data were normalized for individual variation in intracranial vault volume, which was estimated using Brain Extraction Tool (BET) from the FMRIB Software Library from Oxford University [40].

AM1‘s hippocampal volume fell close to 2 SDs below the mean of controls (Z = −1.8) whereas AM2’s hippocampal volume fell more than 2 SDs below the mean of controls (Z = −6.5). In comparison, the volume of the parahippocampal gyrus (temporopolar cortex, perirhinal, entorhinal, and parahippocampal cortices) was not lower than average for both patients (Z = 2.4 and .3, respectively).

An ANOVA (gender×difference scores×group) conducted to examine the influence of gender on the results of Experiment 2 did not reveal a significant interaction on confidence scores [F [2], [20] = 0.3, P = 0.86], R/K judgments [F [2], [20] = 1.2, P = 0.27], or ROC scores [F [2], [20] = 0.39, P = 0.53].

Stimuli consisted of 120 negatively arousing photos, and 120 neutral photos, selected from the same pool of photos as in Experiment 1 (see Experiment 1, methods).

Participants were presented with 60 neutral photos and 60 emotional photos. On each trial a photo was presented for 1s, after which the participant had 2s to rate the photo for visual complexity, then a fixation cross appeared for 6s. The trials were separated into four blocks of 30 trials each.

Two hours after the encoding session participants were trained at making a “remember”/”know” judgment [34] and were given a recognition test. The 2 hour delay period was chosen instead of the 24h delay period used in Exp 1 to avoid floor effects of overall recognition performance of the amnesics and controls, who were older than the young adults tested in Exp 1. Previous studies have shown that this delay is sufficient to lead to an enhancement of memory with emotion [2], [35]. The recognition test included the presentation of 240 photos; 60 old negatively arousing photos, 60 old neutral photos, 60 new negatively arousing photos, and 60 new neutral photos. Stimuli were presented in a random order on a computer screen. Each trial consisted of the presentation of a photo for 2s. The participant then had unlimited time to indicate whether the photo was new, “remembered”, or “known”, by pressing the appropriate key, and to rate the confidence of their recognition response on a scale from 1 (high confidence new photo) to 6 (high confidence old photo).

## Supporting Information

Table S1 Proportion of confidence responses and remember/know judgments for emotional and neutral photos seen either 5min or 24h prior to recognition test, or new.
(0.03 MB DOC)

Table S2 Proportion of confidence responses and remember/know judgments for old and new, emotional and neutral photos, in the amnesic and control groups.
(0.04 MB DOC)

## References

[1] Ochsner KN (2000). Are affective events richly recollected or simply familiar? The experience and process of recognizing feelings past.. Journal of Experimental Psychology General..

[pone.0001068-Sharot1] Sharot T, Delgado MR, Phelps EA (2004). How emotion enhances the feeling of remembering.. Nature Neuroscience,.

[3] Talarico JM, Rubin DC (2003). Confidence, not consistency, characterizes flashbulb memories.. Psychological Science,.

[4] Dolcos F, LaBar KS, Cabeza R (2005). Remembering one year later: role of the amygdala and the medial temporal lobe memory system in retrieving emotional memories.. Proceedings of the National Academy of Science U S A..

[5] Sharot T, Martorella EA, Delgado MR, Phelps EA (2006). How personal experience modulates the neural circuitry of memories of September 11.. Proceedings of the National Academy of Sciences: USA,.

[6] Cahill L, Babinsky R, Markowitsch HJ, McGaugh JL (1995). The amygdala and emotional memory.. Nature,.

[pone.0001068-McGaugh1] McGaugh JL, Christianson S (1992). Affect, neuromodulatory systems and memory storage.. The Handbook of Emotion and Memory: Research and Theory.

[8] Packard MG, Teather LA (1998). Amygdala modulation of multiple memory systems: Hippocampus and caudate-putamen.. Neurobiology of Learning and Memory,.

[9] McGaugh JL (2000). Memory: a century of consolidation.. Science..

[10] Sharot T, Yonelinas AP (2007). Differential time-dependent effects of emotion on recollective experience and memory for contextual information.. Cognition (in press).

[11] Hamann SB, Cahill L, Squire LR (1997). Emotional perception and memory in amnesia.. Neuropsychology..

[12] Adolphs R, Tranel D, Buchanan TW (2005). Amygdala damage impairs emotional memory for gist but not details of complex stimuli.. Nat Neurosci..

[13] Richardson MP, Strange BA, Dolan RJ (2004). Encoding of emotional memories depends on amygdala and hippocampus and their interactions.. Nature Neuroscience,.

[14] Yonelinas AP, Jacoby LL (1995). The relation between remembering and knowing as bases for recognition: effects of size congruency.. Journal of Memory and Language..

[15] Macmillan NA, Creelman CD (1991). Detection theory: a user's guide..

[16] Atkinson RC, Juola JF, Krantz DH, Atkinson RC, Luce RD, Suppes P (1974). Search and decision processes in recognition memory.. Contemporary developments in mathematical psychology.

[pone.0001068-Mandler1] Mandler G (1980). Recognizing: the judgment of previous occurrence.. Psychological Review,.

[18] Yonelinas AP (2002). The nature of recollection and familiarity: a review of 30 years of research.. Journal of Memory and Language,.

[19] Tulving E (1985). Memory and consciousness.. Canadian Psychology..

[20] Fox E, Russo R, Bowles R, Dutton K (2001). Do threatening stimuli draw or hold visual attention in subclinical anxiety?. Journal of Experimental Psychology: General..

[21] Anderson AK, Phelps EA (2001). Lesions of the human amygdala impair enhanced perception of emotionally salient events.. Nature,.

[22] Kensinger EA, Corkin S (2003). Memory enhancement for emotional words: Are emotional words more vividly remembered than neutral words?. Memory and Cognition,.

[23] Kensinger EA, Corkin S (2004). The effects of emotional content and aging on false memories.. Cognitive, Affective, and Behavioral Neuroscience,.

[24] Brown MW, Aggleton JP (2001). Recognition memory: what are the roles of the perirhinal cortex and hippocampus?. Nat Rev Neurosci,.

[25] Stefanacci L, Suzuki WA, Amaral DG (1996). Organization of connections between the amygdaloid complex and the perirhinal and parahippocampal cortices in macaque monkeys.. Journal of Computational Neurology..

[26] Paz R, Pelletier JG, Bauer EP, Pare D (2006). Emotional enhancement of memory via amygdala-driven facilitation of rhinal interactions.. Nat Neurosci..

[27] Fenker DB, Schott BH, Richardson-Klavehn A, Heinze HJ, Duzel E (2005). Recapitulating emotional context: activity of amygdala, hippocampus and fusiform cortex during recollection and familiarity.. European Journal of Neuroscience..

[28] Zola-Morgan S, Squire LR, Amaral DG (1986). Human amnesia and the medial temporal region: enduring memory impairment following a bilateral lesion limited to field CA1 of the hippocampus.. J Neurosci..

[29] Hodges H, Sowinski P, Fleming P, Kershaw TR, Sinden JD (1996). Contrasting effects of fetal CA1 and CA3 hippocampal grafts on deficits in spatial learning and working memory induced by global cerebral ischaemia in rats.. Neuroscience..

[30] Caine D, Watson JD (2000). Neuropsychological and neuropathological sequelae of cerebral anoxia: a critical review.. J Int Neuropsychol Soc..

[31] Rempel-Clower NL, Zola SM, Squire LR, Amaral DG (1996). Three cases of enduring memory impairment after bilateral damage limited to the hippocampal formation.. J Neurosci..

[32] Vargha-Khadem F, Gadian DG, Watkins KE, Connelly A, Van Paesschen W (1997). Differential effects of early hippocampal pathology on episodic and semantic memory.. Science..

[33] Lang PJ, Bradley MM, Cuthbert BN (1999). International affective picture system (IAPS): Instruction manual and affective ratings..

[34] Rajaram S (1993). Remembering and knowing: two means of access to the personal past.. Memory and Cognition,.

[35] LaBar KS, Phelps EA (2005). Reinstatement of conditioned fear in humans is context dependent and impaired in amnesia.. Behav Neurosci..

[36] Verfaellie M, Koseff P, Alexander MP (2000). Acquisition of novel semantic information in amnesia: effects of lesion location.. Neuropsychologia..

[37] Yonelinas AP, Kroll NE, Quamme JR, Lazzara MM, Sauve MJ (2002). Effects of extensive temporal lobe damage or mild hypoxia on recollection and familiarity.. Nat Neurosci..

[38] Kan IP, Giovanello KS, Schnyer D, Makris D, Verfaellie M (in press). Role of the medial temporal lobes in relational memory: Neuropsychological evidence from a cued recognition paradigm.. Neuropsychologia.

[39] Seidman LJ, Faraone SV, Goldstein JM, Kremen WS, Horton NJ (2002). Left hippocampal volume as a vulnerability indicator for schizophrenia: A magnetic resonance imaging morphometric study of nonpsychotic first-degree relatives.. Archives of General Psychiatry,.

[40] Smith SM (2002). Fast robust automated brain extraction.. Human Brain Mapping,.

