# Supplementary material for: How Emotion Strengthens the Recollective Experience: A Time-Dependent Hippocampal Process
Source: PLoS One. 2007 Oct 31;2(10):e1068. doi: 10.1371/journal.pone.0001068 (PMC2031918; doi:10.1371/journal.pone.0001068)
Supplement: Table S1 — Proportion of confidence responses and remember/know judgments for emotional and neutral photos seen either 5min or 24h prior to recognition test, or new. (0.03 MB DOC) [file pone.0001068.s001.doc]

**Table S1**.

|  | Emotional | | | Neutral | | |
| --- | --- | --- | --- | --- | --- | --- |
|  | 5min | 24hr | New | 5min | 24hr | New |
| 6 (high confidence old) | 0.75 | 0.65 | 0.06 | 0.65 | 0.48 | 0.04 |
| 5 (low confidence old) | 0.09 | 0.12 | 0.06 | 0.12 | 0.18 | 0.04 |
| 4 (guess old) | 0.07 | 0.09 | 0.11 | 0.10 | 0.13 | 0.08 |
| 3 (guess new) | 0.05 | 0.06 | 0.15 | 0.05 | 0.09 | 0.15 |
| 2 (low confidence new) | 0.03 | 0.05 | 0.27 | 0.04 | 0.08 | 0.26 |
| 1 (high confidence new) | 0.02 | 0.02 | 0.35 | 0.03 | 0.04 | 0.43 |
| Remember | 0.50 | 0.42 | 0.05 | 0.42 | 0.31 | 0.03 |
| Know | 0.41 | 0.44 | 0.19 | 0.47 | 0.48 | 0.13 |
