# Supplementary material for: How Emotion Strengthens the Recollective Experience: A Time-Dependent Hippocampal Process
Source: PLoS One. 2007 Oct 31;2(10):e1068. doi: 10.1371/journal.pone.0001068 (PMC2031918; doi:10.1371/journal.pone.0001068)
Supplement: Table S2 — Proportion of confidence responses and remember/know judgments for old and new, emotional and neutral photos, in the amnesic and control groups. (0.04 MB DOC) [file pone.0001068.s002.doc]

Table S2.

|  | Emotional | | | | Neutral | | | |
| --- | --- | --- | --- | --- | --- | --- | --- | --- |
|  | Amnesics | | Controls | | Amnesics | | Controls | |
|  | Old | New | Old | New | Old | New | Old | New |
| 6 (high confidence old) | 0.19 | 0.01 | 0.60 | 0.05 | 0.11 | 0.00 | 0.44 | 0.03 |
| 5 (low confidence old) | 0.22 | 0.06 | 0.10 | 0.04 | 0.13 | 0.03 | 0.13 | 0.02 |
| 4 (guess old) | 0.20 | 0.15 | 0.07 | 0.07 | 0.25 | 0.12 | 0.09 | 0.05 |
| 3 (guess new) | 0.06 | 0.11 | 0.04 | 0.06 | 0.10 | 0.12 | 0.06 | 0.06 |
| 2 (low confidence new) | 0.07 | 0.16 | 0.04 | 0.11 | 0.08 | 0.15 | 0.06 | 0.12 |
| 1 (high confidence new) | 0.25 | 0.51 | 0.15 | 0.67 | 0.33 | 0.58 | 0.22 | 0.72 |
| Remember | 0.15 | 0.02 | 0.46 | 0.03 | 0.06 | 0.01 | 0.24 | 0.01 |
| Know | 0.46 | 0.24 | 0.32 | 0.21 | 0.40 | 0.18 | 0.46 | 0.18 |
